# Ubiquitin Ligase Cbl-b Is Involved in Icotinib (BPI-2009H)-Induced Apoptosis and G1 Phase Arrest of *EGFR* Mutation-Positive Non-Small-Cell Lung Cancer

**DOI:** 10.1155/2013/726375

**Published:** 2013-03-19

**Authors:** Xiaodong Mu, Ye Zhang, Xiujuan Qu, Kezuo Hou, Jian Kang, Xuejun Hu, Yunpeng Liu

**Affiliations:** ^1^Department of Respiratory Medicine, The First Hospital of China Medical University, 155 North Nanjing Street, Heping District, Shenyang 110001, China; ^2^Department of Medical Oncology, The First Hospital of China Medical University, 155 North Nanjing Street, Heping District, Shenyang 110001, China

## Abstract

Epidermal growth factor receptor (EGFR) is one of the most promising targets for non-small-cell lung cancer (NSCLC). Icotinib, a highly selective EGFR tyrosine kinase inhibitor (EGFR-TKI), has shown promising clinical efficacy and safety in patients with NSCLC. The exact molecular mechanism of icotinib remains unclear. In this study, we first investigated the antiproliferative effect of icotinib on NSCLC cells. Icotinib significantly inhibited proliferation of the EGFR-mutated lung cancer HCC827 cells. The IC_50_ values at 48 and 72 h were 0.67 and 0.07 **μ**M, respectively. Flow cytometric analysis showed that icotinib caused the G1 phase arrest and increased the rate of apoptosis in HCC827 cells. The levels of cyclin D1 and cyclin A2 were decreased. The apoptotic process was associated with activation of caspase-3, -8, and poly(ADP-ribose) polymerase (PARP). Further study revealed that icotinib inhibited phosphorylation of EGFR, Akt, and extracellular signal-regulated kinase. In addition, icotinib upregulated ubiquitin ligase Cbl-b expression. These observations suggest that icotinib-induced upregulation of Cbl-b is responsible, at least in part, for the antitumor effect of icotinib via the inhibition of phosphoinositide 3-kinase (PI3K)/Akt and mitogen-activated protein kinase pathways in EGFR-mutated NSCLC cells.

## 1. Introduction

Lung cancer is the leading cause of cancer-related death worldwide [1]. Most patients are diagnosed at an advanced stage (IIIB and IV), which limits the surgical options. Platinum doublet chemotherapy is the major treatment for advanced non-small-cell lung cancer (NSCLC), but the outcome is still unsatisfactory, with an objective response rate of 30%–40% and a median survival time of 8–10 months [2]. Combined chemotherapy with cytotoxic drugs usually leads to severe toxicity, which lowers quality of life. Thus, agents with new mechanisms of action and high antitumor activity but low side effects are urgently needed.

Epidermal growth factor receptor (EGFR; ErbB-1) is as a member of the ErbB growth factor receptor tyrosine kinase (TK) family and is frequently overexpressed in many types of human malignancy [3]. In about 50% or more of cases of NSCLC, EGFR protein expression is detected [4]. The continuous activity of EGFR through mutation or overexpression is negatively correlated with prognosis in many types of human malignancy including NSCLC [5]. The receptor TK activity of EGFR can be activated by multiple ligands, such as EGF. Upon ligand binding, the receptor dimerizes and undergoes autophosphorylation at specific tyrosine residues of the intracellular domain. The phosphorylated tyrosine residues then serve as docking sites for proteins such as growth factor receptor-bound 2 (Grb2), collagen domain protein Shc, and phospholipase C, which, in turn, activate downstream signaling pathways, including phosphoinositide 3-kinase (PI3K)/Akt and mitogen-activated protein kinase (MAPK)/extracellular signal-regulated kinase (ERK), which regulate transcription factors and other proteins involved in biological responses such as proliferation, cell motility, angiogenesis, cell survival, and differentiation [6]. Targeted EGFR drugs are currently used clinically and greatly prolong progression-free survival time.

Icotinib hydrochloride, 4-[(3-ethynylphenyl)amino]-6,7-benzo-12-crown-4-quinazoline hydrochloride, is a novel EGFR-TK inhibitor (EGFR-TKI) [7]. Similar to gefitinib and erlotinib, icotinib inhibited growth of human tumor cell lines that overexpress EGFR (IC_50_ 1 mmol/L for A431 cells) and growth of A431 cells (human epithelial carcinomas) in a nude mouse xenograft model. Moreover, in a tolerance clinical trial implemented by Peking Union Medical College Hospital, icotinib exhibited excellent tolerance among healthy Chinese subjects [8]. The exact molecular mechanism of icotinib remains unclear.

Cbl family of ubiquitin ligases are negative regulators of nonreceptor TK and some activated signaling pathways [9]. The TKB domain of the Cbl family proteins can interact with the p85 subunit of PI3K, resulting in their ubiquitination and degradation [10, 11]. We demonstrated recently that the Cbl family can negatively regulate the activity of PI3K/Akt and MAPK/ERK pathways in cancer cells [12–14]. However, the potential role of the Cbl family in EGFR-TKI-induced antitumor activity, via inhibition of the PI3K/Akt and MAPK pathways in NSCLC, has not been identified.

In the present study, we examined the anticancer effect of icotinib in NSCLC cell lines of differing EGFR status. Icotinib caused G1 phase arrest and increased the rate of apoptosis in HCC827 cells. Further study revealed that icotinib inhibited the phosphorylation of EGFR, Akt, and ERK. In addition, icotinib upregulated ubiquitin ligase Cbl-b expression. These observations suggest that icotinib-induced upregulation of Cbl-b is responsible, at least in part, for the antitumor effect of icotinib, via inhibition of PI3K/Akt and MAPK pathways in EGFR-mutated cells.

## 2. Methods

### 2.1. Reagents and Antibodies

Icotinib was provided by Zhejiang Beta Pharma (China) and was dissolved in 5% DMSO to make 10 mM stock solution, which was kept at −20°C and diluted in RPMI-1640 medium (Gibco BRL, Grand Ysland, NY, USA) when used. Anti-caspase-8 antibody was purchased from Lab Vision Corporation (Fremont, CA, USA). Anti-EGFR, anti-phospho-EGFR, anti-Akt, anti-phospho-Akt, anti-ERK, and anti-phospho-ERK antibodies were purchased from Cell Signaling Technology (Danvers, MA, USA). Anti-caspase-3, anti-poly (ADP-ribose) polymerase (PARP), anti-Cbl-b, anti-cyclin A, anti-cyclin D1, anti-cyclin E, and anti-actin were purchased from Santa Cruz Biotechnology (Santa Cruz, CA, USA).

### 2.2. Cell Culture

Human lung adenocarcinoma cells HCC827 and A549 were obtained from the Type Culture Collection of the Chinese Academy of Sciences (Shanghai, China). The cells were cultured in RPMI-1640 medium containing 10% heat-inactivated fetal bovine serum (FBS), penicillin (100 U/mL), and streptomycin (100 mg/mL) at 37°C under an atmosphere of 95% air and 5% CO_2_. The cells were routinely subcultured every 2-3 days and were all from the logarithmic phase of growth.

### 2.3. Cell Viability Assay

The effect of icotinib on HCC827 and A549 cell proliferation was measured using the 3-(4,5-dimethylthiazol-2-yl)-2,5-diphenyl tetrazolium bromide (MTT) assay. Cells were seeded at a density of 1 × 10^4^ cells/well in 96-well plates and incubated overnight. Then, different concentrations of icotinib were added, and the cells were further incubated for the indicated times. An amount of twenty microliters of MTT solution (5 mg/mL) was added to each well, and the cells were incubated for further 4 h at 37°C. After the removal of the culture medium, the cells were lysed in 200 *μ*L DMSO, and OD_570_ was measured using a microplate reader (Model 550; Bio-Rad Laboratories, Hercules, CA, USA). The following formula was used: cell viability = (OD of experimental sample/OD of untreated group) × 100%.

### 2.4. Flow Cytometry Analysis

Cells were seeded at a density of 3.0 × 10^5^ cells/well in six-well plates, incubated overnight, and exposed to icotinib for 24 h. After fixation in ice-cold 70% ethanol for 12 h, the samples were incubated with 20 *μ*g/mL RNase A at 37°C and 10 *μ*g/mL propidium iodide (PI) for 30 min in the dark. Finally, the samples were evaluated by flow cytometry, and the data were analyzed with CellQuest software (Becton Dickinson, San Jose, CA, USA).

### 2.5. Western Blot Analysis

The cells were treated with icotinib as describe earlier and collected for Western blot analysis. The cells were solubilized in 1% Triton lysis buffer (1% Triton X-100, 50 mM Tris–HCl pH 7.4, 150 mM NaCl, 10 mM EDTA, 100 mM NaF, 1 mM Na_3_VO_4_, 1 mM PMSF, and 2 *μ*g/mL aprotinin) and quantified with the Lowry method. Cell lysate proteins were separated by SDS-PAGE and electrophoretically transferred to a nitrocellulose membrane (Immobilon-P; Millipore, Bedford, MA, USA). The membranes were blocked with 5% skimmed milk in trimethyl benzene sulfonyl tetrazole (TBST) buffer (10 mM Tris–HCl pH 7.4, 150 mM NaCl, 0.1% Tween 20) at room temperature for 2 h and incubated overnight at 4°C with primary antibodies. After secondary antibodies were added for 30 min at room temperature, the proteins were detected with enhanced chemiluminescence reagent (SuperSignal Western Pico Chemiluminescent Substrate; Pierce, Rockford, IL, USA) and visualized with the Electrophoresis Gel Imaging Analysis System (DNR Bio-Imaging Systems, Jerusalem, Israel).

### 2.6. Statistical Analysis

The experiments were repeated at least three times. Data are expressed as the means ± SD. Differences in the results for two groups were evaluated by Student's *t*-test. *P* < 0.05 was considered to be statistically significant. IC_50_ values were calculated by nonlinear regression analysis using GraphPad Prism for Windows version 5.00 (GraphPad Software, San Diego, CA, USA).

## 3. Results

### 3.1. Icotinib Significantly Inhibits EGFR-Mutated NSCLC Cell Proliferation

**Figure 1:**
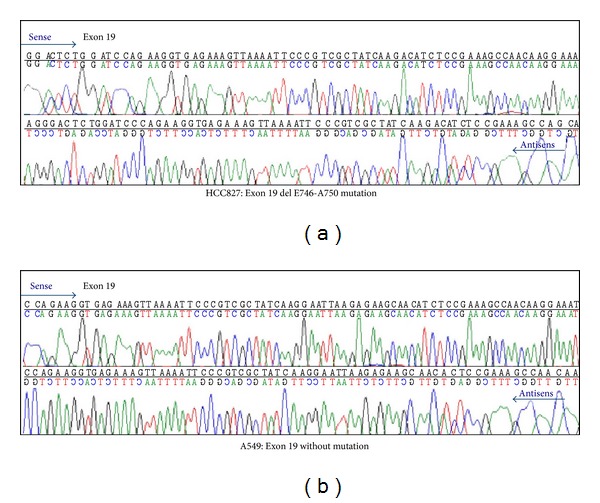
Identification of EGFR mutation in HCC827 and A549 cells. Exon 19 del mutation was detected in the HCC827 cell line.

**Figure 2:**
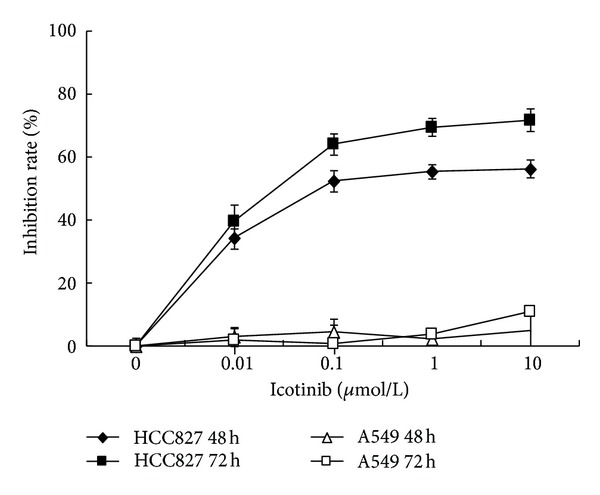
Icotinib inhibited human NSCLC cell proliferation. HCC827 and A549 cells were exposed to icotinib (1 nM–100 *μ*M) for 48 h, and cell viability was determined by MTT assay. Data are the mean ± SD of at least three independent experiments performed in triplicate.

To verify whether icotinib inhibits proliferation of NSCLC cells, HCC827 cells (harboring an EGFR mutation E746-A750del) and A549 cells (EGFR wild type) were exposed to 1 nM to 100 *μ*M icotinib for 48 and 72 h. MTT assays revealed that, in HCC827 cells, the IC_50_ values of icotinib at 48 and 72 h were 0.67 ± 0.33 and 0.07 ± 0.09 *μ*M, respectively. In A549 cells, the IC_50_ of icotinib at 48 and 72 h exceeded 10 *μ*M (Figures 1 and 2).

### 3.2. Icotinib Induces G1 Phase Cell Cycle Arrest and Apoptosis in HCC827 Cells

**Figure 3:**
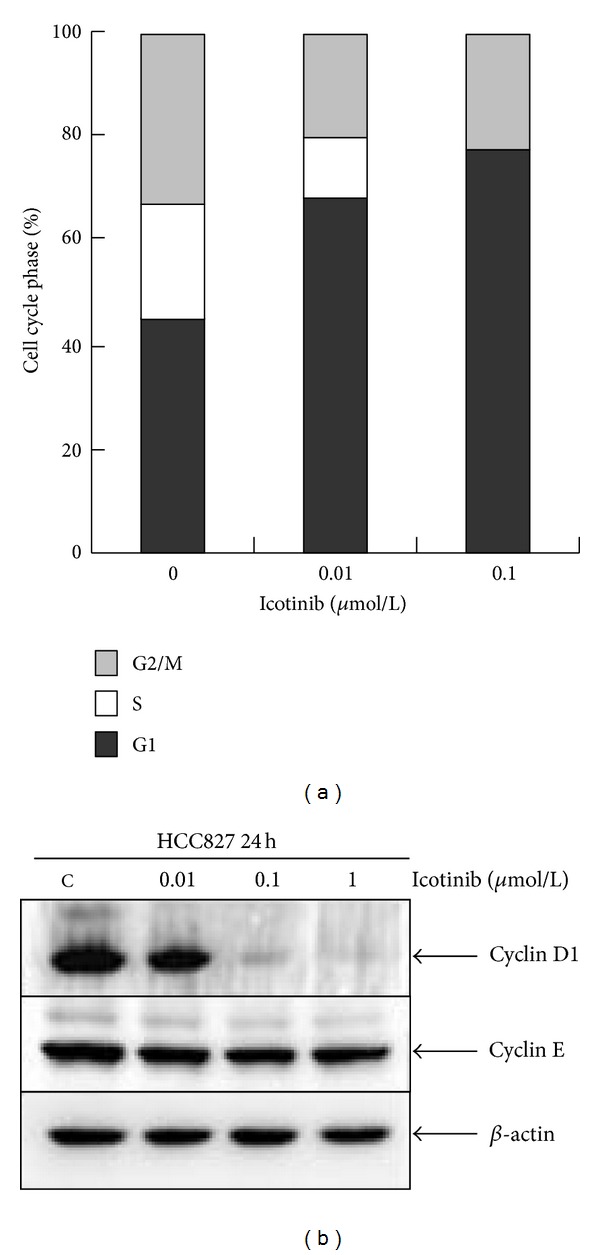
Icotinib-induced G1 phase cell cycle arrest in HCC827 cells. (a) Cells were exposed to 0.01 and 0.1 *μ*mol/L icotinib for 24 h, and the cell cycle was analyzed by flow cytometry after staining with propidium iodide. (b) Expression of cell cycle proteins cyclins D1, A, and E was analyzed by Western blotting. *β*-Actin was used as the internal control. Data are representative of one of three independent experiments.

**Figure 4:**
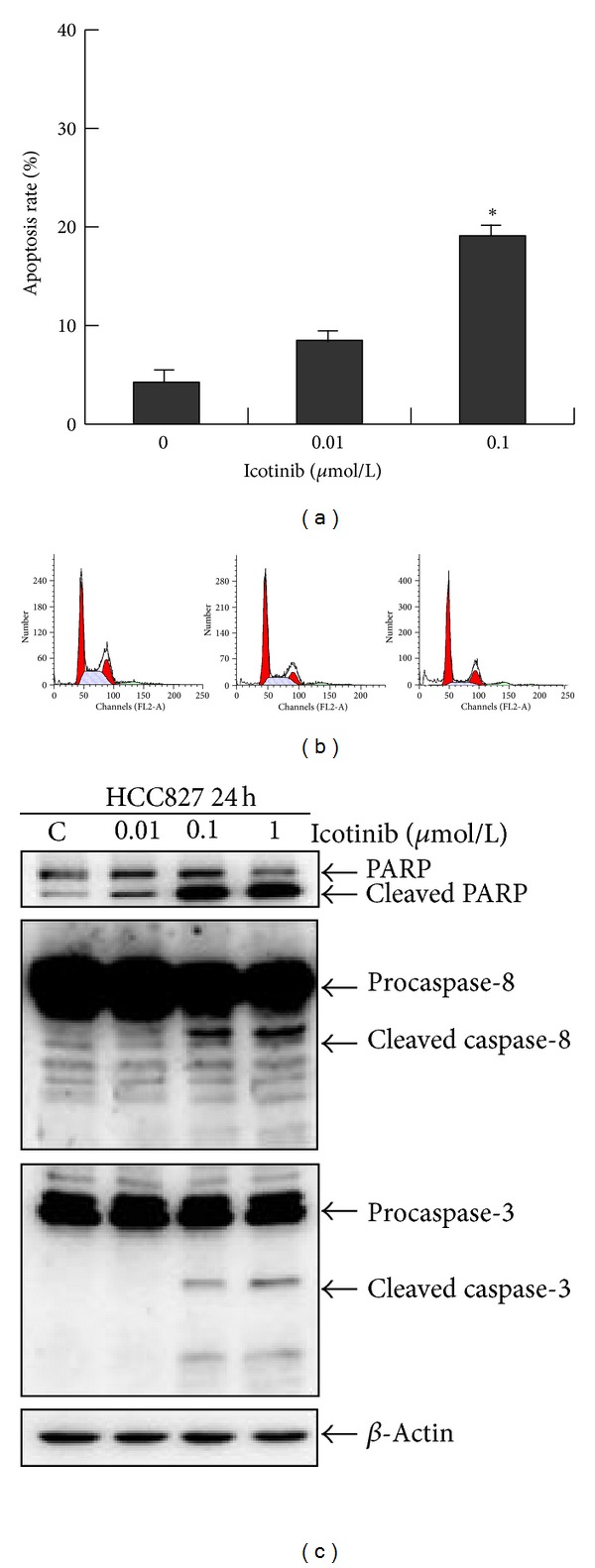
Icotinib-induced apoptosis in HCC827 cells. (a) and (b) Cells were exposed to 0.01 and 0.1 *μ*mol/L icotinib for 24 h, and the cell cycle was analyzed by flow cytometry after staining with propidium iodide. (c) Expression of PARP and caspase-3 and -8 was analyzed by Western blotting. *β*-Actin was used as the internal control. Data are representative of one of three independent experiments.

There was an increase in the number of cells in G1 phase after exposure to 0.01 and 0.1 *μ*M icotinib for 24 h. The percentage of cells in G1 phase increased from 45.41 ± 1.25 to 68.41 ± 2.46 and 78.30 ± 3.02, respectively (Figure 3(a)). Furthermore, the percentages of apoptotic cells were elevated from 4.26 ± 2.98% to 6.31 ± 1.49% and 18.85 ± 1.29%, after treatment with 0.01 and 0.1 *μ*M icotinib (Figures 4(a) and 4(b)).

We further investigated the mechanism of icotinib involved in regulation of cell cycle arrest and apoptosis. Expression levels of cyclin D1, cyclin A, and cyclin E and activation of caspase-3, -8, and PARP were elucidated by Western blotting. Treatment of HCC827 cells with 0.01, 0.1, and 1 *μ*M icotinib for 24 h resulted in decreased expression of cyclin D1 and cyclin A in a dose-dependent manner, with no changes in the levels of cyclin E (Figure 3(b)). Meanwhile, the amount of cleaved caspase-3, -8, and PARP (substrate of caspase-3) increased in a dose-dependent manner (Figure 4(c)). These results showed that icotinib caused significant G1 phase arrest and increased the rate of apoptosis in EGFR-mutated lung cancer cells.

### 3.3. Icotinib Inhibits Activation of EGFR, MAPK, and PI3K Pathways in HCC827 Cells

**Figure 5:**
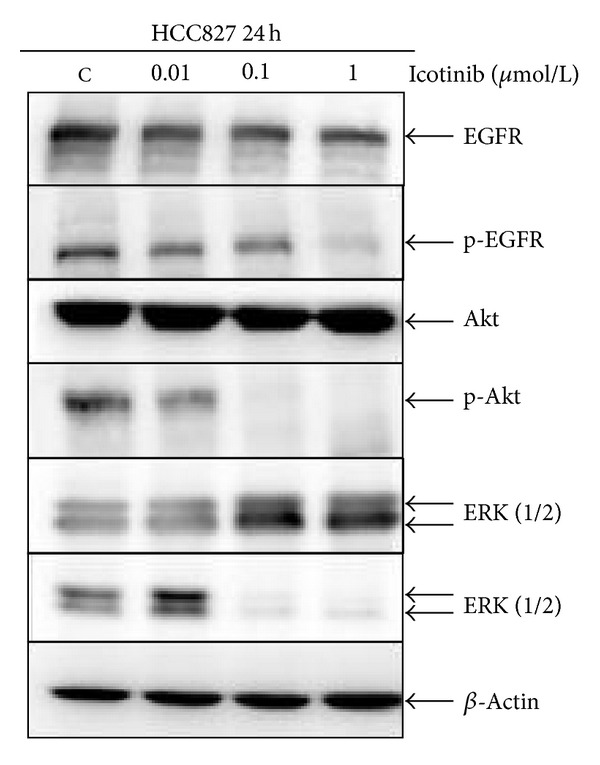
Icotinib regulated phosphorylation of EGFR, Akt, and ERK. Western blot analysis of EGFR, Akt, and ERK protein expression in HCC827 cells treated with 0.01–1 *μ*mol/L icotinib. *β*-Actin was used as the internal control.

To determine whether activation of EGFR, PI3K/Akt, or MAPK/ERK signaling pathways is involved in icotinib-induced cell cycle arrest and/or apoptosis in NSCLC cells, we detected activation of EGFR, Akt, and ERK in HCC827 cells treated with 0.01, 0.1, and 1 *μ*M icotinib for 24 h. Western blotting showed that expression of p-EGFR, p-Akt, and p-ERK proteins was clearly decreased after being treated with icotinib (Figure 5). These data indicate that the PI3K/Akt and MAPK/ERK signaling pathways play a crucial role in regulating icotinib-related cell cycle arrest and apoptosis in HCC827 cells.

### 3.4. Icotinib Upregulates Expression of Cbl-b in HCC827 Cells

**Figure 6:**
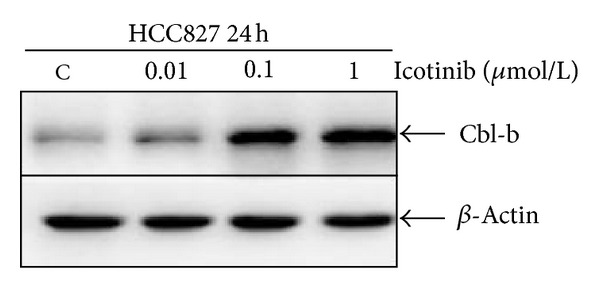
Icotinib upregulated expression of Cbl-b. Cells were exposed to 0.01–1 *μ*mol/L icotinib for 24 h, and expression of Cbl-b proteins was analyzed by Western blotting. *β*-Actin was used as the internal control.

To investigate whether inactivation of p-EGFR, p-Akt, and p-ERK proteins by icotinib is correlated with the Cbl family of ubiquitin ligases, we investigated the expression of Cbl-b. After treatment with 0.01, 0.1, and 1 *μ*M icotinib for 24 h, the expression of Cbl-b was increased by 1.36-, 5.12-, and 6.58-fold, respectively, compared with that in the untreated control group (Figure 6). 

## 4. Discussion

Targeting the EGFR therapies gradually to become new treatment approaches for *EGFR* mutation-positive NSCLC has led to the development of novel agents that inhibit EGFR [5, 15]. Icotinib is as a potent small-molecular inhibitor of EGFR-TKI, shows positive clinical antitumor activity in advanced NSCLC patients, especially those with EGFR mutations, and is approved by the State Food and Drug Administration of China (SFDA) [8, 16].

In the present study, we first investigated the anticancer effect of icotinib in NSCLC cell lines of differing EGFR status. The data indicate that icotinib significantly inhibited proliferation of HCC827 (E746-A750del) but not A549 (wild type) cells. We showed that icotinib triggered cell cycle G1 phase arrest in HCC827 cells. Gefitinib induces significant G1/S blockade in ER-positive breast cells [17], G0/G1 arrest together with G2/M block in pancreatic cancer cells [18], and block cell cycle progression at G1 phase in sensitized hepatocellular carcinoma cells [19]. This may be attributed to the different regulatory mechanism of cell cycle progress in different types of tumor cells. We detected expression levels of cyclin D1 and cyclin E. There was a prominent dose-dependent decline in cyclin D1 immunoblot expression in posttreatment HCC827 cells, but not for cyclin E. This could be because cyclins D1 and E control two different events, which are both rate limiting for the G1/S phase transition [20]. 

Apoptosis is a process of programmed cell death, and most anticancer drugs function primarily to induce apoptosis [21–23]. In the present study, we demonstrated that icotinib-treated HCC827 cells had a significantly higher rate of apoptosis than control cells. We further investigated the mechanism of icotinib-induced apoptosis in HCC827 cells. Icotinib increased expression of activated caspase-3 and -8. The classical caspase substrate PARP was also shown to be cleaved. These findings clearly demonstrated the involvement of the caspase-dependent pathway in icotinib-induced apoptotic death of HCC827 cells.

The Ras/Raf/MEK/ERK (MAPK) and PI3K/PTEN/Akt (PI3K) cascades are two major signaling pathways, which are often activated by genetic alterations in upstream signaling molecules such as EGFR-TKs [6, 24]. We investigated the effect of icotinib on the EGFR signaling pathways by Western blot analysis. Icotinib treatment gradually and persistently inhibited EGFR, ERK, and Akt phosphorylation rather than downregulating protein expression in HCC827 cells, indicating that icotinib may suppress components of the MAPK and PI3K signaling pathways, thereby significantly inhibiting tumor cell growth and inducing apoptosis in sensitive cell lines.

Recent data have implicated the Cbl family of proteins, c-Cbl and Cbl-b, as negative regulators of several signal transduction pathways through their E3 ubiquitin ligase activity [25–27]. They can influence the balance between proliferation and apoptosis by mediating related protein degradation [28–30]. Recent studies have suggested that Cbl family proteins enhance apoptosis through different signaling pathways. c-Cbl promotes T cell receptor-induced thymocyte apoptosis by activating the PI3K/Akt pathway [31]. Our study also reported that interferon-*α* enhances apoptosis induced by tumor necrosis factor-related apoptosis-inducing ligand (TRAIL) in gastric cancer cells, at least partially via downregulation of c-Cbl, and subsequent upregulation of the MAPK/ERK pathway [32]. Our results demonstrated that icotinib upregulated expression of E3 ubiquitin ligase Cbl-b, a homolog of c-Cbl. These observations suggest that icotinib-induced upregulation of Cbl-b is responsible, at least in part, for the antitumor effect of icotinib via the inhibition of PI3K/Akt and MAPK pathways in EGFR-mutated NSCLC cells.

## 5. Conclusions

Our findings support the proposal that icotinib inhibits the proliferation of human lung cancer cells with EGFR mutations by inducing cell cycle arrest and cell death. The anticancer effect of icotinib was associated with inhibition of the PI3K/Akt and MAPK/ERK signaling pathways, and the Cbl family of ubiquitin ligases might be involved in regulation of icotinib-induced apoptosis. The results indicate the potential of icotinib for treating advanced NSCLC patients who harbor activating mutations in the EGFR gene.
